# Supplementary material for: Drivers and Barriers Influencing Adherence to the Mediterranean Diet: A Comparative Study across Five Countries
Source: Nutrients. 2024 Jul 25;16(15):2405. doi: 10.3390/nu16152405 (PMC11313691; doi:10.3390/nu16152405)
Supplement: Supplementary file 1 [file nutrients-16-02405-s001.zip › nutrients-3073055-supplementary.pdf]

PROMEDLIFE Questionnaire

[Language preference]

Please select your preferred language:

- Arab
- Greek
- French
- Italian
- Slovenian

[Brief introduction to the questionnaire]

Dear Participant,

we would like to invite you to take part in a research study involving the administration of a questionnaire with questions concerning certain socio-demographic and lifestyle aspects (dietary habits and preferences, physical activity) conducted as part of the PRIMA European project "PROMEDLIFE".

Before you decide to participate in this study, it is important that you have all the information you need to participate in an informed and responsible manner. We ask you to read the following and to contact Prof. Davide Menozzi (davide.menozzi@unipr.it) with any questions you may have, indicating "PROMEDLIFE STUDY" in the subject line of the email.

Participation is completely voluntary and all information collected will be kept private and confidential. Data from the study are collected anonymously and will only be used for research purposes.

Brief Description and Objectives. *The research project aims to investigate lifestyle (dietary habits and preferences, physical activity) in relation to socio-demographic characteristics.*

What does participation in the study entail? *Participation in this research project entails the honest and sincere fulfilment of an online questionnaire about your socio-demographic and lifestyle characteristics.*

Benefits, inconveniences and/or potential risks of participation. *Participation in the study does not entail any kind of risk or inconvenience.*

Withdrawal from the study*. You have the right to withdraw your consent to participate in this study at any time, even without prior notice or specific reason.*

Return*. You have the right to request information on the results and outcome of the search.*

Measures envisaged to protect anonymity*. The data will be collected anonymously and used exclusively for scientific and statistical purposes and in compliance with current data protection*

legislation. The research results will be published in summary form and under no circumstances will any brief quotations be attributable to individual persons.

Contact. *Please contact Prof. Davide Menozzi (davide.menozzi@unipr.it) for any further information or clarification you may require.*

1. If you agree to these terms and conditions, please select 'I agree' to continue the survey.

- I agree → question n.3
- I don't agree

2. You stated to not consent with the participation terms and do not want to participate in the survey. Are you sure?[1]

- Yes (screen out)
- No, I want to try again

[Screening questions]

3. Please indicate where you live

- Italy (for Italian survey only)
- Greece (for Greek survey only)
- Slovenia (for Slovenian survey only)
- Tunisia (for Tunisian survey only)
- Morocco (for Moroccan survey only)
- Other (screen out)

4. Please indicate how long have you been living in Italy/Greece/Slovenia/Tunisia/Morocco.

- < 1 year[2] (screen out)
- 1-5 years
- >5 years

5. What is your age[3]**?** (A drop-down menu will be shown):

- <18 years (screen out)
- …
- >79 years (screen out)

6. Are you responsible for food purchases for your family or yourself[4]**?**

- Always
- Often
- Sometimes

---

[1] Henn et al, 2022 (doi: 10.1016/j.foodqual.2021.104455)
[2] The idea is to limit the survey to those residing from at least one year in the country of residence for food habits adaptation
[3] Definition of adults based on "adult"[Mesh] in Pubmed excluding "aged, 80 and over"[Mesh].
[4] This screening condition is defined in the Grant Agreement.

- Never (screen out)

[General food-related questions]

7. Are you responsible for meal preparation for your family or yourself?

- Always
- Often
- Sometimes
- Never

8. Do you consider yourself…

- Omnivore (general food regimen, both plants and animals are included)
- Lacto-vegetarian (meat, poultry, fish, eggs are excluded; dairy products such as milk, cheese, yoghurt, and butter are included).
- Ovo-vegetarian (meat, poultry, fish, and dairy products are excluded; eggs are included).
- Lacto-ovo vegetarian (meat, poultry, and fish are excluded; dairy products and eggs are included).
- Pescatarian (meat, poultry, dairy products, and eggs are excluded; fish is included).
- Vegan (meat, poultry, fish, eggs and dairy products are excluded)
- Flexitarian (a plant-based diet with the occasional inclusion of animal products)
- Others (please specify) ______________

9. Please indicate if your food habits are affected by any religious beliefs and/or ethical concerns

- Yes, they are affected by religious beliefs
- Yes, they are affected by ethical concerns
- Yes, they are affected by religious beliefs and ethical concerns
- No, they are not affected by any religious beliefs and/or ethical concerns

10. Are you currently on a low-calorie diet for weight loss or weight loss maintenance?

- Yes, under the supervision of a healthcare professional
- Yes, without the supervision of a healthcare professional
- No
- Other (please specify)________________________

11. How often do you eat meals (breakfast, lunch or dinner) out of home*?

*This includes, for example, meals eaten in restaurants, canteens or self-service restaurants and not meals cooked at home and eaten at work.

- Never
- Less than once a month
- Once a month
- 2-3 times per month
- 1-2 times per week

- 3-4 times per week
- 5 or more times per week

12. How often do you eat fast foods?

- Never
- Less than once a month
- Once a month
- 2-3 times per month
- 1-2 times per week
- 3-4 times per week
- 5 or more times per week

[Food choice motives]

Please answer to the following items on a 7-point scale (1 – Not at all important; 7 – Extremely important)[5]

It is important to me that the food I eat on a typical day…

		1	2	3	4	5	6	7
	**[Health]**							
1.	Contains a lot of vitamins and minerals							
2.	Keeps me healthy							
3.	Is nutritious							
	**[Convenience]**							
4.	Is easy to plan, buy and prepare							
5.	Can be cooked very easily							
6.	Takes very little or no time to prepare							
	**[Sensory appeal]**							
7.	Smells nice							
8.	Has a pleasant texture							
9.	Tastes well							
	**[Natural content]**							
10.	Contains no additives							
11.	Contains natural ingredients							
12.	Contains no artificial ingredients							
	**[Price]**							
13.	Is not expensive							
14.	Is cheap							

[5] Adapted version by Pieniak et al, 2009 (http://dx.doi.org/10.1016/j.appet.2009.05.019).

15.	Is good value for money							
	**[Weight control]**							
16.	Is low in calories							
17.	Helps me control my weight							
18.	Is low in fat							
	**[Familiarity]**							
19.	Is what I usually eat							
20.	Is familiar							
21.	Is like the food I ate when I was a child							
	**[Ethical concern]**							
22.	Is produced using ethical production methods (e.g. sustainable, animal friendly, without child labour, etc.)							
23.	Supports the local economy							
24.	Is produced/packaged in an environmentally friendly way							

[Sustainable food choice motives][6]

Please answer to the following items on a 7-point scale (1 not at all important, 7 Extremely important).
It is important to me that the food I eat on a typical day…

	[General sustainability]	**1**	**2**	**3**	**4**	**5**	**6**	**7**
	[Animal welfare]							
1.	Is produced without animals being in pain							
2.	Is produced in an animal friendly way							
3.	Is produced in Halal way							
	[Ethical concern]							
1.	Is produced without exploitation							
2.	Is produced without child labour							
	[Environmental welfare]							
1.	Is prepared in an environmentally friendly way							
2.	Is produced in an environmentally friendly way							
	[Local and seasonal]							
1.	Is a local/regional product							
2.	Is a seasonal product							
3.	Comes from close by (little transport distance)							

[6] Verain et al, 2021, doi: 10.1016/j.foodqual.2021.104267 (short version used)

[Attitudes toward healthfulness of foods][7]

Please indicate to what extent you agree or disagree with each of the following statements on a 7-point scale: 1 "Strongly disagree" to 7 - Strongly agree".

	**[General health interest -** interest in eating healthily]	**1**	**2**	**3**	**4**	**5**	**6**	**7**
1.	The healthiness of food has little impact on my food choices. **[R]**							
2.	I am very particular about the healthiness of food I eat.							
3.	I eat what I like and I do not worry much about the healthiness of food. **[R]**							
4.	It is important for me that my diet is low in fat.							
5.	I always follow a healthy and balanced diet.							
6.	It is important for me that my daily diet contains a lot of vitamins and minerals.							
7.	The healthiness of snacks makes no difference to me. **[R]**							
8.	I do not avoid foods, even if they may raise my cholesterol. **[R]**							

[Picky eating]

Please indicate to what extent the following statements apply to you (1 - Never; 2 – Rarely; 3 – Sometimes; 4 – Often; 5 – Always).

	**[Meal presentation_1]**	**1**	**2**	**3**	**4**	**5**
1.	I have a strong preference toward specific food presentation					
	**[Food variety_1]**					
2.	I eat a limited number of items from each food group					
	**[Meal disengagement_1]**					
3.	I am often disengaged/uninvolved when sitting at the table for mealtime					
	**[Taste aversion_1]**					
4.	I reject bitter foods, even if they are only slightly bitter					
	**[Meal presentation_2]**					
5.	I prefer foods of a particular color					
	**[Food variety_2]**					
6.	My usual diet lacks a variety of food groups					
	**[Meal disengagement_2]**					
7.	I usually feel that I have something better to do than eating					
	**[Taste aversion_2]**					
8.	I reject sour foods					
	**[Meal presentation_3]**					
9.	I cringe, cry, or gag after seeing or eating certain foods					
	**[Food variety_3]**					

[7] Roininen et al, 1999 (doi: 10.1006/appe.1999.0232)

10.	I do not like trying new foods					
	**[Meal disengagement_3]**					
11.	I avoid mealtimes					
	**[Meal presentation_4]**					
12.	I am sad or disappointed when food is not prepared/cooked in the "right way".					
	**[Food variety_4]**					
13.	I eat from a very narrow range of foods (<10 different foods).					
	**[Meal presentation_5]**					
14.	I am immediately suspicious of food and feel the need to carefully inspect the majority of food.					
	**[Meal presentation_6]**					
15.	I eat foods in a specific sequence.					
	**[Meal presentation_7]**					
16.	I will not eat a food if I saw someone else touch it.					
	**[Taste aversion_3]**					
17.	I prefer textures that are hard, dry or crunchy					
	**[Taste aversion_4]**					
18.	I avoid foods that are mushy or slippery					
	**[Taste aversion_5]**					
19.	I prefer only pureed or smooth foods					
	**[Taste aversion_6]**					
20.	I prefer only sweet or salty foods that do not have a significant amount of other flavors					

[Questionnaire to evaluate the adherence to the Mediterranean Diet – MEDAS questionnaire as used by Turki et al, 2022[8]]

Please answer to the following statements.

	**Questions**	**Alternative answers** (the score is not shown to the participant)
1.	Do you use olive oil as your main culinary fat?	○ Yes **[1 point]**<br>○ No
2.	How many teaspoons of olive oil do you consume in a given day (including that used for frying, salads, out-of-house meals, etc.)?	○ 4 or more **[1 point]**<br>○ 2 or 3<br>○ Less than 1
3.	How many portions of vegetables (cooked and raw potatoes and beans are not included) do you eat per day? (One serving = 200 g = half a large plate)	○ 3 or more **[1 point]**<br>○ 1 or 2 **[1 point]**<br>○ Less than 1
4.	How many servings of fresh fruit (including natural fruit juices) do you consume per day? (Serving size = one unit of medium-sized fruit, one large cup of sliced fruit, one slice of medium-sized melon/watermelon, or one cup of fresh juice)	○ 3 or more **[1 point]**<br>○ 1 or 2<br>○ Less than 1
5.	How many servings of red meat (veal, beef, mutton, pork[9])/ hamburgers/other meat products (salami, merguez, sausage…) do you consume per day? (One serving = 100 to 150 g = one quarter to half of a meal dish)	○ Less than 1 **[1 point]**<br>○ 2 to 4<br>○ 5 to 6<br>○ 7 or more
6.	How many portions of butter, margarine or cream do you consume per day? (One portion = 12 g = one dessert spoon for butter and margarine, 2 tablespoons for cream)	○ Less than 1 **[1 point]**<br>○ 1<br>○ More than 1
7.	How much sugary drinks (industrial juices)/carbonated drinks/sodas do you consume per day? (One serving equals 330 mL)	○ Less than 1 **[1 point]**<br>○ 1<br>○ More than 1
8.	How many glasses/cups of wine do you consume per week?[10]	○ More than 14 glasses (more than 2 glasses per day) **[1 point]**<br>○ 7 to 14 glasses (1 or 2 glasses per day) **[1 point]**<br>○ 2 to 6 glasses (sometimes but not every day)<br>○ One glass or less (occasionally)

[8] Turki, S., Bouzekri, K., Trabelsi, T., & El Ati, J. (2022). Assessment of Mediterranean Diet Adherence and Lifestyle Change during COVID-19 National Lockdown in Tunisian Adult Population. Nutrients, 14(19), 4151.
[9] Only in Italy, Greece and Slovenia.
[10] Not included in Tunisia and Morocco.

		○ None
9.	How many portions of legumes (lentils, beans, chickpeas, peas…) do you consume per week? (One portion = 150 g = one dish or glass)	○ 3 or more **[1 point]**<br>○ 1 to 2<br>○ Less than 1
10.	How many portions of fish and seafood do you eat per week? (One portion = 100 to 150 g = a quarter to half of a meal dish)	○ 3 or more **[1 point]**<br>○ 1 to 2<br>○ Less than 1
11.	How many times a week do you consume industrial (not homemade) desserts/confectionery/pastries? (Including cakes, biscuits, ice cream, etc.)	○ Less than 1 **[1 point]**<br>○ 1 to 2 **[1 point]**<br>○ 3 or more
12.	How many servings of nuts (unsalted) do you consume per week? (Including unsalted peanuts, almonds, hazelnuts, walnuts, etc. One serving = 30 g = one handful)	○ 3 or more **[1 point]**<br>○ 1 to 2<br>○ Less than 1
13.	Do you prefer to consume chicken, turkey, or rabbit meat, or a vegetarian protein source, rather than red meat or derived products?	○ Yes **[1 point]**<br>○ No
14.	How many times a week do you eat dishes cooked with tomatoes or tomato sauce, onion and/or garlic and olive oil?	○ Twice or more **[1 point]**<br>○ Once or less

[Questionnaire to evaluate drivers and barriers to the Mediterranean Diet][11]

This figure[12] represents the Mediterranean Diet, a dietary pattern linked to the traditional food styles of countries bordering the Mediterranean Sea. Preference for local, seasonal, fresh and minimally processed food is emphasised, supporting biodiversity and eco-friendly and traditional foods. This dietary pattern is based on the consumption of:

· numerous plant foods (fruit, vegetables, bread, other cereals, potatoes, legumes);
· olive oil as the main source of fat;
· milk products and dairy products (mainly cheese and yogurt);
· fish and poultry in moderate quantities;
· up to four eggs per week;
· an extremely reduced quantity of red meat;
. an occasional consumption of sweets
· a moderate quantity of wine mainly during meals.

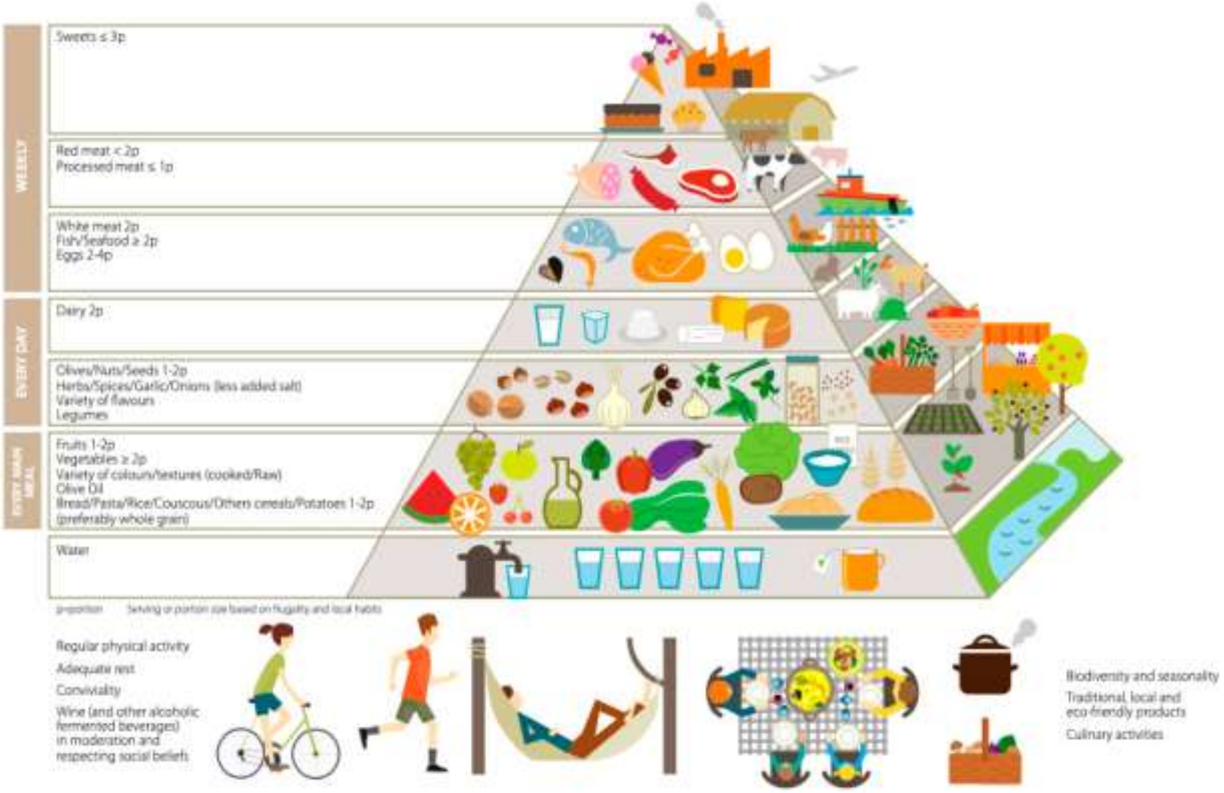

11 Scannell et al, 2020 (doi:10.3390/ijerph17249321)
12 Serra-Majem et al, 2020, doi: 10.3390/ijerph17238758.

Please indicate to what extend according to you the following statements are true or fault, on a 7 point scale (1 = Strongly disagree – 7 = Strongly agree)

DRIVERS								
		**1**	**2**	**3**	**4**	**5**	**6**	**7**
**[HEALTH]**								
1.	Mediterranean Diet has a positive effect on cholesterol.							
2.	Mediterranean Diet lowers LDL (bad) cholesterol levels.							
3.	Mediterranean Diet reduces the risk of cardiovascular disease, diabetes, mental illness, depression, cancer, and obesity.							
**[DIET QUALITY]**								
4.	Mediterranean Diet includes healthier and more nutritious foods.							
5.	Mediterranean Diet is defined by a higher fruit and vegetable consumption and lower red meat consumption.							
6.	Mediterranean Diet includes more beneficial oils for health.							
**[APPLICABILITY]**								
7.	Mediterranean Diet is tastier and more sustainable than other types of diets.							
**[LIFESTYLE]**								
8.	Mediterranean Diet increases consumption of homemade foods.							
9.	Mediterranean Diet includes more unprocessed and additive-free foods.							
10.	Mediterranean Diet is associated with higher socialization and family relationships.							
**[AFFORDABILITY]**								
11.	Food access is easier in Mediterranean Diet.							
12.	Mediterranean Diet contains lower-priced foods.							
**[ENVIRONMENT]**								
13.	Mediterranean Diet has a positive effect on the environment.							
14.	Mediterranean Diet reduces human impact on the environment.							
15.	Mediterranean Diet is associated with better carbon footprint.							
16. F	To demonstrate that you are not a robot, for this statement, please select "Strongly agree"							
17.	Mediterranean Diet contains more local foods.							
BARRIERS								
**[HEALTH]**								

18.	Mediterranean Diet contains more allergenic foods.							
19.	Mediterranean Diet is not applicable for vegans.							
20.	Food variety in Mediterranean Diet is insufficient.							
21.	Preparing meals suitable for Mediterranean Diet is difficult and time-consuming.							
22.	Mediterranean Diet is restrictive.							
23.	Mediterranean Diet contains unpleasant-tasting foods.							
24.	It is difficult to diversify food recipes in Mediterranean Diet.							
**[LIFESTYLE]**								
24.	Following Mediterranean Diet is difficult due to conflict with cultural habits/beliefs/norms.							
**[AFFORDABILITY]**								
25.	Mediterranean Diet contains high-priced foods.							
26.	There are limited options in shops for foods in Mediterranean Diet.							
27.	There are limited options in restaurants for foods in Mediterranean Diet.							

[Attitude towards the MD][13]

Following the Mediterranean Diet for me would be:

Please indicate your opinion using the following 7-point scale.

1. Disgusting - □ □ □ □ □ □ □ - Tasty
2. Unpleasant - □ □ □ □ □ □ □ - Pleasant
3. Disadvantageous - □ □ □ □ □ □ □ - Advantageous
4. Useless – □ □ □ □ □ □ □ - Useful

[Clinical questions]

1. **Please indicate if you have any medically diagnosed chronic disease.**
   - ○ Yes
   - ○ No

2. **Please indicate if you have any food intolerances or allergies**
   - ○ Yes
   - ○ No

[13] See Carfora et al, 2022, doi:10.1017/S1368980022000878.

3. **To your knowledge, do you have vitamins and/or minerals deficiencies (e.g., iron, vitamin B12, vitamin D etc.)**
   - Yes
   - No

4. **Do you take any food supplements? (e.g., iron, vitamin B12, vitamin D, omega 3, etc.)**
   - Yes
   - No

5. **Please indicate your smoking habits**[14]
   - Never
   - I quit
   - Sometimes
   - Usually

10. Please indicate your weight (in kg) (a drop-down menu will be shown) ________________

11. Please indicate your height (in cm) (a drop-down menu will be shown)_______________

[Food insecurity Experience Scale - FIES]

During the last 12 months, was there a time when:

		Yes	No	I Don't know	I refuse to answer
	**[WORRIED]**				
1	You were worried you would not have enough food to eat because of a lack of money or other resources.				
	**[HEALTHY]**				
2	Still thinking about the last 12 months, was there a time when you were unable to eat healthy and nutritious food because of a lack of money or other resources				
	**[FEW FOODS]**				
3	You ate only a few kinds of foods because of a lack of money or other resources				
	**[SKIPPED]**				
4	You had to skip a meal because there was not enough money or other resources to get food				
	**[ATE LESS]**				
5	Still thinking about the last 12 MONTHS, was there a time when you ate less than you thought you should because of a lack of money or other resources				
	**[RUN OUT]**				

[14] Menghi et al, 2022 (doi: 10.1016/j.foodqual.2022.104647).

6	Your household ran out of food because of a lack of money or other resources					
	**[HUNGRY]**					
7	You were hungry but did not eat because there was not enough money or other resources for food					
	**[WHOLE DAY]**					
8	During the last 12 MONTHS, was there a time when you went without eating for a whole day because of a lack of money or other resources?					

[Socio-demographic questions]

1. Please indicate your sex:

- Female
- Male go to question n. 3a/b/c/d/e
- Other go to question n. 3a/b/c/d/e
- Prefer not to answer go to question n. 3a/b/c/d/e

2. Please indicate if you are:

- Pregnant
- Breastfeeding
- In perimenopause (<12 consecutive months from the last menstruation)
- In menopause (≥ 12 consecutive months from the last menstruation)
- None of the above

3a. Please indicate your geographical area of residence (Italy)

- North-West (Piemonte, Valle d'Aosta, Liguria e Lombardia)
- North-East (Emilia-Romagna, Veneto, Trentino-Alto Adige, Friuli Venezia Giulia)
- Centre (Toscana, Marche, Umbria, and Lazio)
- South (Abruzzo, Molise, Campania, Puglia, Calabria, and Basilicata)
- Islands (Sicilia and Sardegna)

3b.Please indicate your geographical area of residence (Greece)

- Attica / Αττική
- Central Greece / Στερεά Ελλάδα
- Central Macedonia / Κεντρική Μακεδονία
- Crete / Κρήτη
- Eastern Macedonia and Thrace / Ανατολική Μακεδονία και Θράκη
- Epirus / Ήπειρος
- Ionian Islands / Ιόνια νησιά
- North Aegean / Βόρειο Αιγαίο
- Peloponnese / Πελοπόννησος
- South Aegean / Νότιο Αιγαίο
- Thessaly / Θεσσαλία

- Western Greece / Δυτική Ελλάδα
- Western Macedonia / Δυτική Μακεδονία
- Monastic community of Mount Athos / Άγιον Όρος

3c.Please indicate your geographical area of residence (Slovenia)

- Eastern Slovenia (Vzhodna Slovenija – including the Mura, Drava, Carinthia, Savinja, Central Sava, Lower Sava, Southeast Slovenia, and Littoral–Inner Carniola regions).
- Western Slovenia (Zahodna Slovenija – including the Central Slovenia, Upper Carniola, Gorizia, and Coastal–Karst regions)

3d.Please indicate your geographical area of residence (Morocco)

- Northwest (Rabat-Salé-Zemmour-Zaer, Gharb-Chrarda-Béni Hssen, Tangier-Tetouan)
- Eastern (Oriental)
- Centre-North (Fès-Boulemane, Taza-Al Hoceima-Taounate)
- Centre (Grand Casablanca, Chaouia-Ouardigha, Tadla-Azilal)
- Centre-South (Meknès-Tafilalet)
- South (Souss-Massa-Drâa, Guelmim-Es Semara, Laâyoune-Boujdour-Sakia El Hamra, Oued Ed-Dahab-Lagouira)
- Tensfit (Marrakesh-Tensift-El Haouz, Doukkala-Abda)

3e1.Please indicate in which geographical area you live (Tunisian Governorates)

- North East (Bizerte, Tunis, Aryanah, Menouba, Ben Arous, Zaghouan and Nabeul)
- North West (Béja, Kef, Siliana and Jendouba)
- Center East (Monastir, Mahdia, Sfax and Sousse)
- Center West (Sidi Bouzid, Kairouan and Kasserine)
- South East (Gabes, Medenine and Tataouine)
- South West (Gafsa, Kebili and Tozeur)

4. Please choose from the following the type of area you live in:[15]

- Large urbanization area (more than 1.5 million inhabitants)
- Metropolitan area (between 500,000 and 1.5 million inhabitants)
- Medium-sized urban area (between 200,000 and 500,000 inhabitants)
- Small urban area (between 50,000 and 200,000 inhabitants)
- Nearby rural area (<50,000 inhabitants but less than 60 minutes by car from a center of at least 50,000 inhabitants)
- Remote rural area (<50,000 inhabitants but more than 60 minutes by car from a center of at least 50,000 inhabitants)

---

[15] Definition retrieved from FAO, 2018 (https://www.fao.org/3/ca6392en/ca6392en.pdf) and from Childs et al, 2022 (doi: 10.34172/hpp.2022.02).

5. Please indicate your educational attainment[16]

- Not attending school
- Primary school license
- Lower secondary school diploma (middle school diploma)
- Secondary school diploma (secondary school diploma)
- Diploma of technical or professional institution
- Bachelor's degree, 1st level academic diploma/school for linguistic mediators
- One-cycle degree, Master's Degree
- Post-graduate degree (e.g., PhD, Medical Specialisation Schools etc.)
- Other:____

6. Please indicate your occupation[17]

- Unemployed
- Student
- Full-time worker (mostly sedentary work)
- Full-time worker (mostly dynamic, non-sedentary job)
- Part-time worker (mostly sedentary job)
- Part-time worker (mostly dynamic, non-sedentary job)
- Retired

7. Please indicate your level of income[18] (average monthly income of your family)

- A lot of difficulties getting to the end of the month
- Some difficulty getting to the end of the month
- No difficulty in reaching the end of the month
- Manage to save money every month
- I refuse to answer

8. How many people (including you) are part of your household?

- 1 (only me)
- 2
- 3
- 4
- 5
- >5

---

[16] Greece: https://gpseducation.oecd.org/Content/MapOfEducationSystem/GRC/GRC_2011_EN.pdf
Italy: https://it.wikipedia.org/wiki/Classificazione_internazionale_standard_dell%27istruzione
Morocco: https://education-profiles.org/northern-africa-and-western-asia/morocco/~morocco
Slovenia: https://www.eurydice.si/publikacije/The-Education-System-in-the-Republic-of-Slovenia-2018-19.pdf
Tunisia: https://education-profiles.org/northern-africa-and-western-asia/tunisia/~tunisia

[17] We identify two categories of workers to differentiate the office workers from those with a manual/factory type of job.

[18] From Sgroi et al, 2022, doi: 10.1016/j.jafr.2022.100371

9. How many people under the age of 18 are in your household?

- None
- 1
- 2
- 3
- 4
- 5
- >5

[Physical activities in your free time][19]

The following questions concern how physically active you are in your free time and during transport (including your commute to and from work)

[MVPA question]

1. On a typical week, how much time do you spend in total on moderate and vigorous physical activities where your heartbeat increases and you breathe faster (e.g. brisk walking, cycling as a means of transport or as exercise, heavy gardening, running, or recreational sports).

Only include activities that lasted at least 10 minutes at a time.

- Less than ½ an hour (less than 30 minutes) per week (go to 3)
- ½ an hour - 1 ½ hour (30-90 minutes) per week (go to 2.1)
- 1 ½ - 2 ½ hours (90-150 minutes) per week (go to 2.2)
- 2 ½ - 5 hours (150-300 minutes) per week (go to 2.3)
- More than 5 hours (more than 300 minutes) per week (go to 2.3)

[VPA question]

2.1. How much of the time that you spend on physical activities in a typical week, which you indicated above, do you spend in total on vigorous physical activities? This includes activities that get your heart racing, make you sweat, and leave you so short of breath that speaking becomes difficult (e.g., swimming, running, cycling at high speeds, cardio training, weightlifting, or team sports such as football).

Only include activities that lasted at least 10 minutes at a time.

- Less than ½ an hour (less than 30 minutes) per week
- ½ an hour - 1 hour (30-60 minutes) per week
- 1 - 1 ½ hour (60-90 minutes) per week

2.2. How much of the time that you spend on physical activities in a typical week, which you indicated above, do you spend in total on vigorous physical activities? This includes activities

[19] https://bmcpublichealth.biomedcentral.com/articles/10.1186/s12889-018-5538-y#Sec22

that get your heart racing, make you sweat, and leave you so short of breath that speaking becomes difficult (e.g., swimming, running, cycling at high speeds, cardio training, weightlifting, or team sports such as football).

Only include activities that lasted <u>at least</u> 10 minutes at a time.

- Less than ½ an hour (less than 30 minutes) per week
- ½ an hour - 1 hour (30-60 minutes) per week
- 1 - 1 ½ hour (60-90 minutes) per week
- 1 ½ - 2 ½ hours (90-150 minutes) per week

2.3. How much of the time that you spend on physical activities in a typical week, which you indicated above, do you spend in total on vigorous physical activities? This includes activities that get your heart racing, make you sweat, and leave you so short of breath that speaking becomes difficult (e.g., swimming, running, cycling at high speeds, cardio training, weightlifting, or team sports such as football).

Only include activities that lasted <u>at least</u> 10 minutes at a time.

- Less than ½ an hour (less than 30 minutes) per week
- ½ an hour - 1 hour (30-60 minutes) per week
- 1 - 1 ½ hour (60-90 minutes) per week
- 1 ½ - 2 ½ hours (90-150 minutes) per week
- More than 2 ½ hours (more than 150 minutes) per week

[Survey end]

The survey has ended, thank you for participating in the research, your input is valuable.

[Only for the pre-test]

If you have any suggestions or comments to improve the survey, please write them in the box below.

